# ORMDL3 Functions as a Negative Regulator of Antigen-Mediated Mast Cell Activation *via* an ATF6-UPR-Autophagy–Dependent Pathway

**DOI:** 10.3389/fimmu.2021.604974

**Published:** 2021-02-19

**Authors:** Jia Li, Md Ashik Ullah, Hongping Jin, Yuting Liang, Lihui Lin, Juan Wang, Xia Peng, Huanjin Liao, Yanning Li, Yiqin Ge, Li Li

**Affiliations:** ^1^ Department of Laboratory Medicine, Shanghai General Hospital, Shanghai Jiao Tong University School of Medicine, Shanghai, China; ^2^ Respiratory Immunology Laboratory, QIMR Berghofer Medical Research Institute, Brisbane, QLD, Australia; ^3^ Department of Cell and Molecular Biology, QIMR Berghofer Medical Research Institute, Brisbane, QLD, Australia; ^4^ Center of Clinical Laboratory, The First Affiliated Hospital of Soochow University, Suzhou, China

**Keywords:** orosomucoid-like 3, mast cell activation, degranulation, activating transcription factor 6, autophagy, passive cutaneous anaphylaxis

## Abstract

Antigen (Ag)-mediated mast cell activation plays a critical role in the immunopathology of IgE-dependent allergic diseases. Restraining the signaling cascade that regulates the release of mast cell-derived inflammatory mediators is an attractive therapeutic strategy to treat allergic diseases. Orosomucoid-like-3 (ORMDL3) regulates the endoplasmic reticulum stress (ERS)-induced unfolded protein response (UPR) and autophagy. Although ERS/UPR/autophagy pathway is crucial in Ag-induced mast cell activation, it is unknown whether ORMDL3 regulates the ERS/UPR/autophagy pathway during mast cell activation. In this study, we found that ORMDL3 expression was downregulated in Ag-activated MC/9 cells. Overexpression of ORMDL3 significantly inhibited degranulation, and cytokine/chemokine production, while the opposite effect was observed with ORMDL3 knockdown in MC/9 cells. Importantly, ORMDL3 overexpression upregulated mediators of ERS-UPR (SERCA2b, ATF6) and autophagy (Beclin 1 and LC3BII). Knockdown of ATF6 and/or inhibition of autophagy reversed the decreased degranulation and cytokine/chemokine expression caused by ORMDL3 overexpression. Moreover, *in vivo* knockdown of ORMDL3 and/or ATF6 enhanced passive cutaneous anaphylaxis (PCA) reactions in mouse ears. These data indicate that ORMDL3 suppresses Ag-mediated mast cell activation *via* an ATF6 UPR-autophagy dependent pathway and thus, attenuates anaphylactic reaction. This highlights a potential mechanism to intervene in mast cell mediated diseases.

## Introduction

Mast cells are the key effector cells inducing immunoglobulin E (IgE)-mediated inflammatory responses to allergens in sensitized individuals (1). Mast cells express high-affinity FcϵRI which binds to antigen (Ag)-specific IgE resulting in mast cell sensitization. Upon subsequent exposure to the specific antigen, these sensitized mast cells undergo degranulation and release histamine and lipid mediators (prostaglandins, leukotrienes) followed by a diverse range of cytokines and chemokines (2). These inflammatory mediators trigger acute allergic reactions as observed in allergic disorders, such as allergic asthma, atopic dermatitis, allergic rhinitis, and life-threatening anaphylaxis (3). Hence, a better understanding of the regulatory mechanisms of mast cell activation and subsequent release of inflammatory mediators and how this can be restrained to restore homeostasis is critical for the identification of novel therapeutic targets to treat mast cell mediated diseases.

Orosomucoid-like 3 (*ORMDL3*) gene was first identified in 2007 as an asthma risk gene (4). To date, a number of studies have been performed to investigate the molecular mechanisms by which ORMDL3 contributes to the pathogenesis of asthma (5–7). Airway epithelial cells overexpressing ORMDL3 showed increased transcriptions of genes encoding matrix metalloproteinase, chemokine and CXC chemokine (IL-8, CXCL-10 and CXCL-11), oligoadenylate synthetases (OAS), and ATF6 (8). Overexpression of ORMDL3 in bone marrow-derived eosinophils causes increased rolling, distinct cytoskeletal rearrangement and nuclear translocation of nuclear factor kappa B. Knockdown of ORMDL3 significantly inhibits activation-induced eosinophils shape changes, adhesion and recruitment to sites of inflammation *in vivo* (9). In yeast ORMDL proteins control sphingolipid biosynthesis by regulating the bioactivity of serine palmitoyl transferase (SPT), the rate-limiting enzyme of *de novo* pathway (10). However, the regulatory role of mammals ORMDL proteins in lipid metabolism appears to be much more complicated. Kiefer and his colleagues demonstrated that mammalian SPT activity seems to be affected only when simultaneously enhancing the expression of ORMDL1, 2, and 3 while solo manipulation of any member had no effect (11–13). As an endoplasmic reticulum (ER)-resident transmembrane protein, ORMDL3 also regulates ER stress (ERS) and unfolded protein response (UPR) (10, 14). UPR is comprised of three major signaling pathways, which are initiated by the activation of three protein sensors—activating transcription factor 6 (ATF6), inositol—requiring enzyme 1α (IRE1α) and PKR—like ER kinase (PERK). All three arms of UPR regulate autophagy (15–19). Multiple studies have attempted to uncover the physiological role of ORMDL3 in the cells involved in allergic asthma including airway epithelial cells, eosinophils, macrophages and B cells (8, 9, 20, 21). ORMDL3 specifically binds to and inhibits the sarcoendoplasmic reticulum calcium ATPase (SERCA) 2b resulting in reduction of ER Ca^2+^ concentration and activation of ERS-induced UPR signaling in HEK293 cells (12, 22). Conversely, ORMDL3 has been shown to increase ATF6α level and subsequent induction of SERCA2b expression in human bronchial epithelial cells (BEC) (8), suggesting ORMDL3 mediated ERS-UPR response is cell-specific.

ORMDL3 negatively regulates mast cell activation (23), with ORMDL3 expression found to be lower in Ag-activated mast cells, without affecting the degranulation process. However, the molecular mechanism by which ORMDL3 regulates mast cell function remains largely unknown. The high secretory demand of mast cells is largely dependent on a well-developed ER and, consequently, UPR signal (24, 25). Activation of mast cells initiates the onset of dramatic Ca^2+^ mobilization and triggers degranulation (26, 27). Autophagy, a regulatory process of removing and degrading malfunctioning proteins and organelles, and pathogens (28), is also critical for the degranulation of mast cells. Bone marrow-derived mast cells (BMMCs) deficient in the autophagy related gene (Atg)-7 exhibit normal granule formation, but defective IgE-mediated degranulation demonstrating the importance of autophagic machinery in granule movement and release (29). Given the role of ORMDL3 in ERS-UPR and autophagy in different immune/non-immune cells and the requirement of ERS-UPR and autophagy in mast cell degranulation, we hypothesized that ORMDL3 induces ERS-UPR as well as autophagy in mast cells and thus, ORMDL3 regulates mast cell degranulation and cytokine/chemokine responses.

## Materials and Methods

### Antibodies and Reagents

Antibodies against ORMDL3, ATF6, XBP1, p-eIF2α, SERCA2 ATPase, LC3B, and Beclin 1 were purchased from Abcam (Cambridge, MA, USA). FITC-Concanavalin A was obtained from MKbio (Shanghai, China). 3-MA was purchased from Sigma-Aldrich (St. Louis, MO, USA).

### Cell Culture and Treatment

The MC/9 mouse mast cells (ATCC CRL-8306) were cultured in DMEM supplemented with 10% FBS, 0.05 mM 2-mercaptoethanol, 0.1 mM MEM non-essential amino acids, 100 U/ml penicillin, 100 µg/ml streptomycin, 2 mM L-glutamine, 10 ng/ml recombinant murine IL-3 and 10 ng/ml recombinant murine SCF at 37°C with 5% CO_2_. To inhibit autophagy, cells were serum starved overnight, then treated with 3-MA (Sigma-Aldrich, USA) at indicated concentrations for 24 h. LC3B expression was measured in cell lysates by western blot to confirm the inhibitory effect of 3-MA.

### Vector Construction

To construct the overexpression vector of ORMDL3, mouse *ORMDL3* gene coding sequence was synthesized according to the gene sequence (NM_025661) in the GenBank and inserted into the vector pLenti-GFP-IRES (provided by Novobio Shanghai, China) *via* NheI and AscI restriction endonuclease sites. To generate the knockdown vector of ORMDL3, shRNA was prepared by synthesizing and annealing two oligonucleotides (Forward primer 5’-CACCGCCAAGTATGACCAAGTCCATTCGA AAATGGACTTGGTCATACTTGG-3’ and Reverse complementary primer 5’-AAAACCAAGTATG ACCAAGTCCATTTTCGAATGGACTTGGTCATACTTGGC-3’) and then cloned into the vector pLenti-U6-shRNA-GFP (provided by Novobio Shanghai, China) *via* two BsmBI sites. The knockdown vector of ATF6 was constructed by using designed shRNA oligonucleotides (Forward primer 5’-CCGGGCACTTTGATGCAGCACATGACGAATCATGTGCTGCATCAAAGTGCTTTTT-3’ and Reverse complementary primer 5’-AATTAAAAAGCACTTTGATGCAGCACATGATTCGTCATGT GCTGCATCAAAGTGC-3’) which were then inserted into an inducible knockdown system pLKO-Tet-On (Addgene 21915) *via* AgeI and EcoRI sites. All the constructs were verified by Sanger sequencing.

### Virus Like Particles Production

HEK293T cells were seeded in a 10-cm dish (5 × 10^6^ cells) 1 day before transfection. The vectors of ORMDL3 overexpression, ORMDL3-shRNA and ATF6-shRNA were respectively transfected into HEK293T cells together with lentiviral packaging plasmids pSPAX2 (Addgene 12260) and pMD2G (Addgene 12259) at a ratio of 10 µg: 10 µg: 5 µg by using linear polyethylenimine (PEI). Six hours after transfection, media was changed to fresh DMEM with 5% FBS plus Pen/Strep. Forty-eight hours after transfection, VLPs in the supernatant were harvested by filtration with a sterile 0.45 μm filter and stored in -80°C. VLPs were quantified by using CAp24 ELISA.

### Cell Transduction

MC/9 cells were plated at a density of 2 × 10_4_ cells per 35 mm plate and transduced with VLPs (equivalent to 50 ng of CAp24) conveying ORMDL3, ORMDL3-shRNA or ATF6-shRNA with Polybrene at a concentration of 8 μg/ml. Transduced cells were selected with 2.5 µg/ml blasticidin S (Sigma-Aldrich, MO, USA). To induce varying levels of ATF6 downregulation, 10 ng/ml or 100 ng/ml of doxycycline was added in the cell culture. The transduction results were obtained using fluorescence microscope 48 h post-transduction. The transduction efficiency was assessed by measuring mRNA levels using qRT-PCR and protein expression using Western blot.

### Assessment of Mast Cell Degranulation

MC/9 cells were sensitized overnight with 1 µg/ml of anti-DNP mouse IgE (SPE-7 monoclonal, Sigma, St. Louis, USA) and then washed three times with PBS. Cells were stimulated with the indicated concentrations of DNP–BSA (Santa Cruz, CA, USA) for 30 min. The supernatants were collected and cell pellets were lysed with 0.5% Triton-X 100 at 37°C for 30 min. Commercial ELISA kits were used to detect the concentrations of histamine (Elabscience, Wuhan, China), β-glucuronidase (Elabscience, Wuhan, China) and tryptase (Cusabio, Wuhan, China) in both supernatant and cell lysates. The percentage released was calculated using the following formula: release (%) = [S/(S + L)] × 100, where S and L refer to the concentrations in supernatant and cell lysate, respectively.

### Quantitative Real-Time PCR

Total RNA was isolated from cells using TRIZOL reagent following manufacturer’s protocols (Invitrogen, Carlsbad, CA, USA). Reverse transcription was performed using the SuperScript III Reverse Transcriptase (Invitrogen, Carlsbad, CA, USA) as per manufacturer’s instructions. qRT-PCR reaction was performed using the ChamQ SYBR qPCR Master Mix (Vazyme, China) on a CFX96™ Real-Time System. The data was normalized to the level of β-actin gene expression in the cell samples and calculated as a fold change of the corresponding control. All qRT-PCR primer sequences are listed in **Supplementary Table 1**.

### Western Blot Analysis

Total protein (30 µg) was separated from cell lysates by SDS-PAGE at 100 V for 90 min and transferred onto a PVDF membrane (Bio-Rad, Hercules, CA). After blocking with 5% non-fat milk in TBS containing 0.01% Tween-20 (TBST) at room temperature for 1 h, the membrane was incubated with primary antibodies diluted at 1:1000 (Antibodies against ORMDL3, ATF6, XBP1, p-eIF2α, SERCA2 ATPase, LC3B, and Beclin 1, Abcam, USA) overnight at 4°C. After washing with TBST, the membrane was incubated with HRP-conjugated secondary antibodies diluted at 1:2000 in 5% non-fat milk at room temperature for 1 h. After washing with TBST three times, immunoreactive bands were detected using an ECL Kit (Thermo Scientific, MA, USA) and visualized using a ChemiDoc MP imager (Bio-Rad, California, USA) according to the manufacturer’s instructions. Protein levels were normalized to the amount of GAPDH used as a loading control and to the corresponding controls.

### Confocal Microscopy

For ORMDL3, ATF6 and LC3B staining, treated MC/9 cells were first fixed with 4% paraformaldehyde for 30 min and permeabilized using 0.5% Triton X-100 in PBS for 10 min. Non-specific binding was blocked with 10% normal goat serum in PBS and then incubated with anti-ORMDL3 (1:100 dilution; Abcam, USA), anti-ATF6 (1:100 dilution; Abcam, USA) or anti-LC3B (1:100 dilution; Abcam, USA) overnight. Following labeling with corresponding secondary antibodies (1:1000 dilution; iFluor 555 goat anti-rabbit IgG (H+L), AAT Bioquest, CA, USA) for 1 h at room temperature, the cells were counterstained using DAPI (1:10,000 dilution; Sigma, USA) and imaged on a Super-resolution Multiphoton Confocal Microscope (Leica, Germany). For ER staining, cells were incubated with FITC labelled Concanavalin A (5 µg/ml; MKbio, China) at room temperature for 30 min after DAPI.

### Passive Cutaneous Anaphylaxis

All experiments and animal care procedures conform to the Guide for the Care and Use of Laboratory Animals and were approved by the Animal Ethics Committee of Shanghai General Hospital (No. 2016KY246). Eight-week-old BALB/c mice were injected into both ears (intradermally) with 20 μl of PBS containing different combinations of ORMDL3 overexpressing VLPs (20 ng of CAp24), ORMDL3 knockdown VLPs (20 ng of CAp24) and ATF6 knockdown VLPs (20 ng of CAp24) as follows: ORMDL3-OE group was given ORMDL3 overexpression VLPs; ORMDL3-KD group was given ORMDL3 knockdown VLPs; ATF6-KD group was given ATF6 knockdown VLPs; ORMDL3-OE + ATF6-KD group was given ORMDL3 overexpression VLPs and ATF6 knockdown VLPs; ORMDL3-KD + ATF6-KD group was given ORMDL3 knockdown VLPs and ATF6 knockdown VLPs. Negative and positive control groups were not given any VLPs. Forty-eight hours later, 20 μl PBS containing 100 ng anti-DNP IgE (SPE-7 monoclonal, Sigma, St. Louis, USA) was intradermally injected into both ears. The next day, 200 μl PBS containing 10 μg DNP-BSA (Santa Cruz, CA, USA) and 1% Evans blue was injected intravenously, negative control group was given PBS containing Evans blue only. Two hours later, the mice were euthanized and skin areas were photographed. Skin samples were harvested and Evans blue dye was extracted by incubating the samples in 0.5 ml DMSO for 24 h at 37 °C, and optical density (O.D.) was measured at 650 nm. ORMDL3 and ATF6 gene expression were measured by qRT-PCR to confirm the efficiency of overexpression and knockdown strategies.

### Statistical Analysis

Data were presented as mean ± standard deviation (SD). One-way ANOVA or two-way ANOVA with a Turkey’s multiple comparisons test was applied as appropriate. GraphPad Prism 8.2.1 software (La Jolla, CA, USA) was used for statistical analyses. (NS denotes not significant; * denotes *P* < 0.05; ** denotes *P* < 0.01; *** denotes *P* < 0.001; **** denotes *P* < 0.0001).

## Results

### ORMDL3 Is Downregulated in Ag-Activated Mast Cells

**Figure 1:**
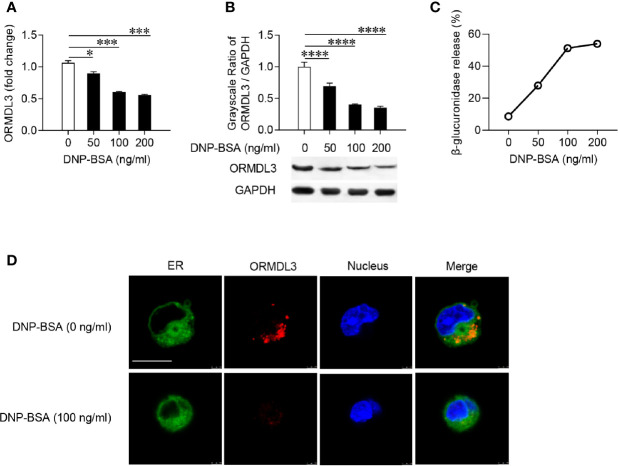
ORMDL3 is downregulated in Ag-activated mast cells. MC/9 cells were sensitized overnight with 1 µg/ml of anti-DNP mouse IgE followed by stimulation with PBS (0 ng/ml DNP-BSA) or indicated concentrations of DNP-BSA for 30 min. **(A)** Expression of ORMDL3 mRNA was measured by qRT-PCR. Data were normalized to the amount of β-actin and calculated as a fold change of the non-stimulated group. **(B)** Expression of ORMDL3 protein was measured by western blotting. GAPDH served as a loading control. **(C)** The release of β-glucuronidase was determined by ELISA. **(D)** Representative images depict ORMDL3 expression [red, stained with iFluor 555 goat anti-rabbit IgG (H+L)] in MC/9 cells. Endoplasmic reticulum (ER, green) was stained with FITC-Concanavalin A and nuclei (blue) were stained with DAPI. Scale bar 10 μm. All the results are shown as mean ± SDs of three independent experiments. *P < 0.05; ***P < 0.001; ****P < 0.0001.

Firstly, mouse MC/9 mast cells were sensitized with DNP-specific IgE and activated with DNP-BSA at different concentrations. The expression of ORMDL3 in mast cells was measured by qRT-PCR and western blot. Ag-activation dose-dependently suppressed ORMDL3 expression at both mRNA and protein levels in MC/9 cells with the maximum response observed with the challenge of DNP-BSA at 100 ng/ml (**Figures 1A, B**). This was corroborated by an increase in the release of β-glucuronidase, a marker of mast cell degranulation (**Figure 1C**). Reduction in ORMDL3 expression was not associated with increased death of activated mast cells (data not shown). Next, we stained ER using FITC-Concanavalin A and found that ORMDL3 is localized to the ER in MC/9 cells (**Figure 1D**). Importantly, the expression of ORMDL3 was lower in Ag-activated mast cells (**Figure 1D**). Taken together, this data confirms that Ag-activation downregulates ORMDL3 expression.

### ORMDL3 Negatively Regulates Degranulation and the Production of Cytokines and Chemokines in Ag-Activated Mast Cells

**Figure 2:**
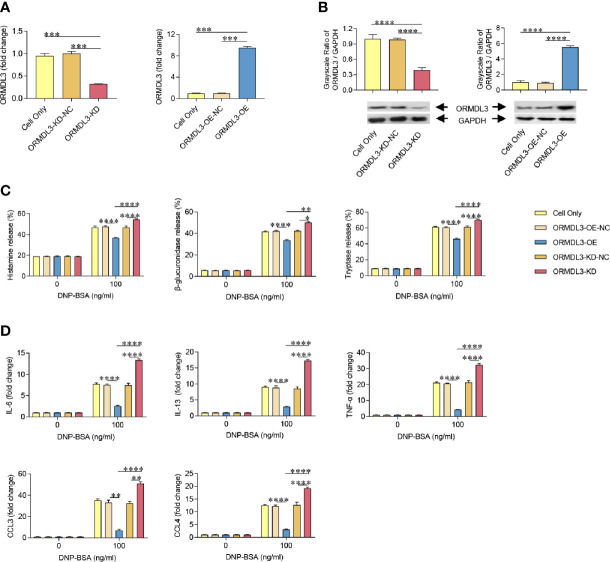
ORMDL3 negatively regulates degranulation and the production of cytokines and chemokines in Ag-activated mast cell. MC/9 cells were transduced with VLPs conveying ORMDL3-shRNA and ORMDL3 and referred to as ORMDL3-KD and ORMDL3-OE respectively. Controls transduced with empty VLPs are referred to as ORMDL3-KD-NC and ORMDL3-OE-NC. **(A, B)** The knockdown and overexpression efficiency were determined by qRT-PCR and western blotting. ORMDL3 mRNA level was normalized to the amount of β-actin and calculated as a fold change of cell only group. For western blotting, GAPDH was used as a loading control. **(C, D)** Cells were sensitized overnight with 1 µg/ml of anti-DNP mouse IgE followed by stimulation with PBS (0 ng/ml DNP-BSA) or 100 ng/ml of DNP-BSA for 30 min. Release of histamine, β-glucuronidase and tryptase was measured by ELISA. Quantification of cytokines (IL-6, IL-13, and TNF-α) and chemokines (CCL3 and CCL4) was performed by qRT-PCR. Data were normalized to the amount of β-actin and calculated as a fold change of the non-stimulated cell only group. Results are shown as mean ± SDs of three independent experiments. *P < 0.05; **P < 0.01; ***P < 0.001; ****P < 0.0001. VLPs, virus like particles; KD, knockdown; OE, overexpression; NC, negative control.

To further investigate the role of ORMDL3 in the activation of mast cells, we generated MC/9 cells with knockdown or overexpression of ORMDL3. ORMDL3 mRNA was knocked down approximately 70% by ORMDL3 shRNA (ORMDL3-KD) in MC/9 cells compared to the negative control shRNA (ORMDL3-KD-NC) and cell only groups (**Figure 2A**). The knockdown level of ORMDL3 protein was confirmed by western blot analysis. The level of ORMDL3 protein in the knockdown cells reduced by approximately 60% when compared to that of the negative control and cell only groups as shown in **Figure 2B**. Overexpression of ORMDL3 (ORMDL3-OE) was conducted by stably expressing ORMDL3 with a lentiviral vector containing ORMDL3 cDNA in MC/9 cells. The increase of ORMDL3 expression was checked at both the mRNA (**Figure 2A**) and protein levels (**Figure 2B**). The mRNA level of ORMDL3 was increased ~10-fold when compared to that of cells expressing the empty vector (ORMDL3-OE-NC) and cell only. The protein level of ORMDL3 increased approximately 6-fold when compared to controls.

Using the cell models established above, we assessed the effects of ORMDL3 on Ag-induced degranulation of MC/9 cells, which was evaluated by the release levels of histamine, β-glucuronidase and tryptase. Antigen induction significantly increased degranulation in the cell only and ORMDL3 controls (ORMDL3-OE-NC and ORMDL3-KD-NC) as evidenced by increased release of histamine, β-glucuronidase and tryptase (**Figure 2C**). Importantly, Ag-induced degranulation was significantly down-regulated by the overexpression of ORMDL3 (ORMDL3-OE) and up-regulated by the knockdown of ORMDL3 (ORMDL3-KD; **Figure 2C**). Consistent with the release results, the concentrations of histamine, β-glucuronidase and tryptase were decreased in the supernatant of Ag-activated cells in the ORMDL3-OE group and increased in the ORMDL3-KD group (**Supplementary Figure 1**). This was coupled with an increase of the intracellular concentrations of these markers in ORMDL3-OE cells and a decrease in ORMDL3-KD cells (**Supplementary Figure 1**).

Mast cells also produce cytokines and chemokines critical for inflammatory responses. Hence, we next measured the expression of cytokines and chemokines in Ag-activated mast cells. Ag-induction promoted the release of key effector cytokines IL-6, IL-13, and TNF-α and chemokines CCL3 and CCL4. Importantly, the release of these cytokines/chemokines were significantly impaired in ORMDL3-OE cells and increased in ORMDL3-KD cells (**Figure 2D**). In the non-activated MC/9 cells, there were no differences in the productions of studied cytokines and chemokines. Collectively, these findings suggest that ORMDL3 is a negative regulator of Ag-activated mast cell degranulation as well as cytokine/chemokine production.

### ORMDL3 Regulates ATF6-UPR and Autophagy in Ag-Activated Mast Cells

**Figure 3:**
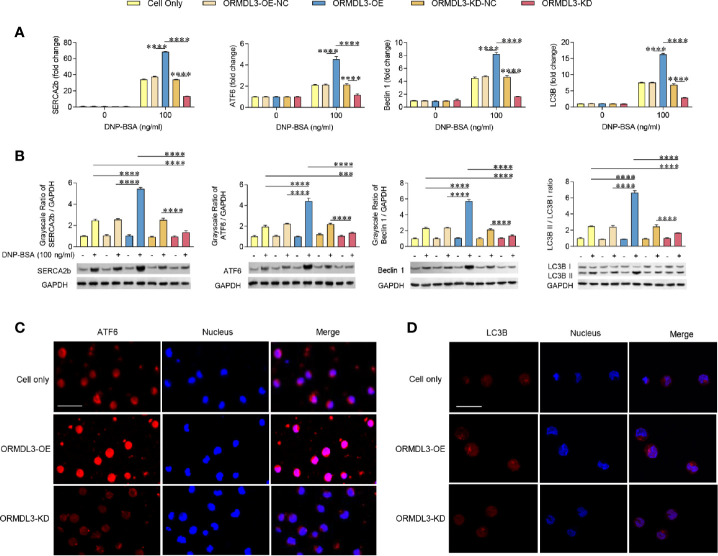
ORMDL3 regulates ATF6-UPR and autophagy in Ag-activated mast cell. MC/9 cells were treated as described in **Figure 2**. mRNA **(A)** and protein **(B)** expression of ERS (SERCA2b, ATF6) and autophagy (Beclin 1, LC3B and LC3B II/LC3B I) markers in non-activated and Ag-activated mast cells was measured by qRT-PCR and western blotting. mRNA level was normalized to the amount of β-actin and calculated as a fold change of the non-stimulated cell only group. For western blotting, GAPDH served as a loading control. **(C, D)** Representative images depict expressions of ATF6 (red) and LC3B (red) in Ag-activated mast cells. Scale bar 25 μm. Nuclei (blue) were stained with DAPI. Results are shown as mean ± SDs of 3 independent experiments. ***P < 0.001; ****P < 0.0001. KD, knockdown; OE, overexpression; NC, negative control.

To investigate whether the ERS-induced UPR-autophagy pathway participates in the ORMDL3-mediated negative regulation of mast cell activation, we compared the ERS/UPR signaling and autophagy biomarkers between ORMDL3-OE and ORMDL3-KD cells. The mRNA levels of ERS markers SERCA2b/ATF6 and autophagy markers Beclin 1 and light chain 3B (LC3B) showed a significant increase in the activated ORMDL3-OE cells and a decrease in ORMDL3-KD cells (**Figure 3A**). The protein levels of SERCA2b, ATF6, Beclin 1 and LC3B II/I also increased in ORMDL3-OE cells and decreased in ORMDL3-KD cells (**Figure 3B**). Other UPR signaling molecules including XBP1u, XBP1s, Perk and BiP were independent of ORMDL3 expression level (**Supplementary Figure 2A**), but the protein level of phosphorylated eukaryotic translation initiation factor 2α (p-eIF2α) was up-regulated in activated ORMDL3-OE cells and down-regulated in ORMDL3-KD cells (**Supplementary Figure 2B**). Confocal microscopy analysis further verified the effects of ORMDL3 on the ATF6-autophagy pathway. As shown in **Figures 3C, D**, overexpression of ORMDL3 led to an increased nuclear localization of ATF6 and a significant increase of LC3B puncta in Ag-activated MC/9 cells, whereas these markers were down-regulated in ORMDL3-KD cells. No significant difference was observed between non-activated groups (**Supplementary Figures 3A, B**).

### Inhibition of Either ATF6 or Autophagy Reverses ORMDL3 Overexpression-Mediated Suppression of Mast Cell Activation

**Figure 4:**
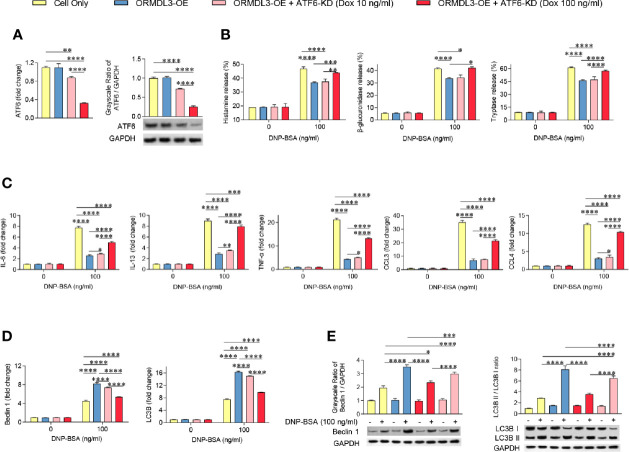
Knockdown of ATF6 reverses ORMDL3 overexpression-mediated suppression of mast cell activation. Knockdown of ATF6 was conducted by transducing ORMDL3-OE cells with VLPs conveying ATF6-shRNA and the addition of doxycycline (Dox, 10 ng/ml or 100 ng/ml). Cells were sensitized overnight with 1 µg/ml of anti-DNP mouse IgE followed by stimulation with PBS (0 ng/ml DNP-BSA) or 100 ng/ml of DNP-BSA for 30 min. **(A)** The knockdown efficiencies were determined by qRT-PCR and western blotting. mRNA level was normalized to the amount of β-actin and calculated as a fold change of the cell only group. For western blotting, GAPDH served as a loading control. **(B)** Release of histamine, β-glucuronidase and tryptase was measured by ELISA. **(C)** mRNA expression of cytokines (IL-6, IL-13, and TNF-α) and chemokines (CCL3 and CCL4). Data were normalized to the amount of β-actin and calculated as a fold change of the non-stimulated cell only group. **(D, E)** qRT-PCR and western blot analysis of autophagy markers (Beclin 1, LC3B, and LC3B II/LC3B I). mRNA level was normalized to the amount of β-actin and calculated as a fold change of the non-stimulated cell only group. For western blotting, GAPDH served as a loading control. Results are shown as mean ± SDs of three independent experiments. *P < 0.05; **P < 0.01; ***P < 0.001; ****P < 0.0001. KD, knockdown; OE, overexpression.

ATF6 is an important protein sensor and is activated during ORMDL3-mediated ERS/UPR signaling. To validate whether ATF6-UPR facilitates ORMDL3-mediated negative regulation of MC/9 function, we adapted a Tet-on system to knockdown the expression of ATF6 to different levels in ORMDL3-OE cells. The result showed that the expression of ATF6 was decreased by doxycycline (Dox) in a dose-dependent manner, which was confirmed by qRT-PCR and western blot analysis (**Figure 4A**). Interestingly, Ag-induced degranulation, and the production of cytokines and chemokines was up-regulated in ATF6-KD (Dox 100 ng/ml) ORMDL3-OE cells, suggesting that impairments of degranulation and cytokine and chemokine production caused by overexpression of ORMDL3 were overcome by ATF6 downregulation, albeit not to the level of un-manipulated cells (**Figures 4B, C**). In line with this, the activated ATF6-KD (Dox 100 ng/ml) ORMDL3-OE cells had higher concentrations in supernatant and lower intracellular concentrations of histamine, β-glucuronidase and tryptase compared with ORMDL3-OE cells (**Supplementary Figure 4**). However, downregulation of ATF6 by Dox at 10 ng/ml did not rescue the impairment of degranulation caused in ORMDL3-OE cells, but it did partially reinitiate the production of IL-6, IL-13, TNF-α and CCL4 (**Figure 4C**). Moreover, as shown in **Figures 4D, E**, the increased Beclin1 and LC3B mRNA expression as well as the increased protein level of Beclin1 and LC3B II/I in ORMDL3-OE cells were markedly reversed by ATF6 knockdown (100 ng/ml and 10 ng/ml Dox), suggesting that autophagy appears to be downstream of the ORMDL3/ATF6 pathway.

**Figure 5:**
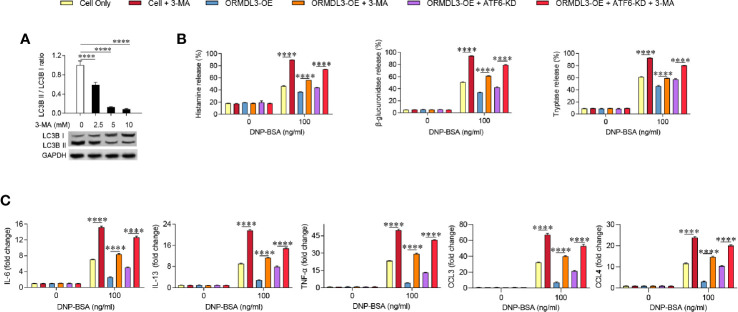
Inhibition of autophagy reverses the effect of ORMDL3 overexpression-induced suppression of mast cell activation. **(A)** MC/9 cells were serum starved overnight, then treated with 3-MA at indicated concentrations for 24 h. Western blot analysis shows the inhibition efficiency of indicated concentrations of 3-MA on LC3B expressions. GAPDH served as a loading control. **(B, C)** ORMDL3 overexpression and ATF6 knockdown were conducted as described in **Figures 2** and **4**. Cells were serum starved overnight followed by treatment with 1 µg/ml of anti-DNP mouse IgE together with or without 5 mM 3-MA for 24 h, then stimulated with 100 ng/ml of DNP-BSA for 30 min. Release of histamine, β-glucuronidase and tryptase was measured by ELISA. Quantification of cytokines (IL-6, IL-13, and TNF-α) and chemokines (CCL3 and CCL4) mRNAs was performed by qRT-PCR. Data were normalized to the amount of β-actin and calculated as a fold change of the non-stimulated cell only group. Results are presented as mean ± SDs of three independent experiments. ****P < 0.0001. KD, knockdown; OE, overexpression.

To further determine the contribution of autophagy in the inhibition role of ORMDL3 on mast cell activation, ORMDL3-OE cells were treated with 3-MA, which is widely used as an inhibitor of autophagy. The inhibition of autophagy was demonstrated by the low expression of LC3B II/LC3B I, which was confirmed by western blot analysis (**Figure 5A**). As shown in **Figures 5B, C**, induction of degranulation and cytokine/chemokine production in the 3-MA treated Ag-activated MC/9 cells was significantly increased when compared to the cell only group. When ORMDL3-OE cells were treated with 3-MA and activated, the decreased degranulation and the production of cytokines and chemokines caused by ORMDL3 overexpression was reversed. A similar result was observed in the 3-MA treated ATF6-KD ORMDL3-OE cells. Taken together, these results suggest that ORMDL3 negatively regulates degranulation as well as cytokine and chemokine production of Ag-activated MC/9 cells *via* the ATF6-autophagy pathway.

### Knockdown of ORMDL3 and/or ATF6 Enhances PCA Reactions

**Figure 6:**
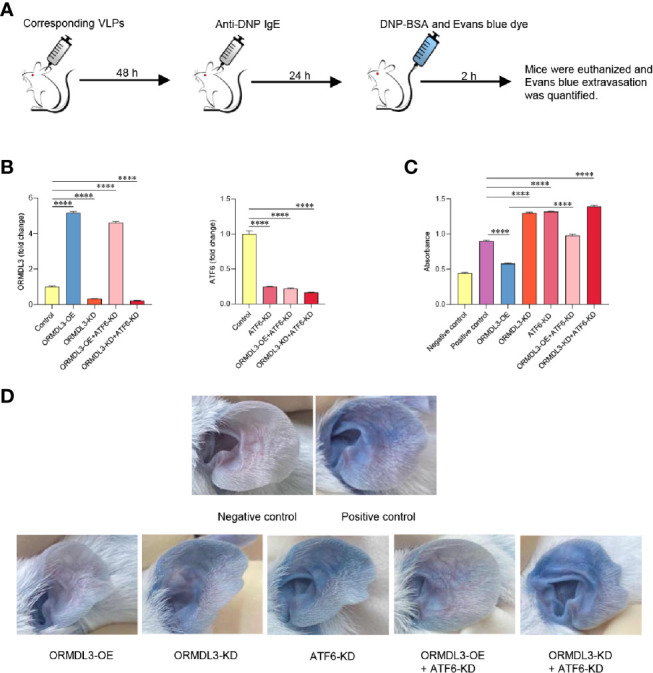
Knockdown of ORMDL3 and/or ATF6 enhances passive cutaneous anaphylaxis (PCA) reactions. **(A)** The ears of mice were injected intradermally with corresponding VLPs (ORMDL3 overexpressing, knockdown and/or ATF6 knockdown); both PCA negative and positive control were VLPs-free. Anti-DNP IgE was injected intradermally into the ears 48 h later. DNP-BSA and Evans blue was injected intravenously another 24 h later, the negative control received Evan blue in PBS only. Two hours later, mice were euthanized. **(B)** ORMDL3 and ATF6 quantification of extracts from ears was performed by qRT-PCR. Data were normalized to the amount of β-actin and calculated as a fold change of the control group. Results are presented as mean ± SDs of 3 independent experiments. **(C)** Evans blue dye was extracted by incubating the ear samples in 0.5 ml DMSO for 24 h at 37 °C, and optical density (O.D.) was measured at 650 nm. Quantitative data are presented as mean ± SDs (n=6; pooled data from two independent experiments). **(D)** Representative photographs of ears from indicated mice 2 h after antigen challenge. ****P < 0.0001. VLPs, virus like particles; KD, knockdown; OE, overexpression.

To evaluate the *in vivo* relevance of the ORMDL3-mediaed ATF6 UPR pathway in mast cell functions, mice were intradermally injected into both ears with ORMDL3 overexpressing virus like particles (VLPs), ORMDL3 knockdown VLPs and/or ATF6 knockdown VLPs to change ORMDL3 and/or ATF6 expression locally (**Figure 6A**). After 48 h of injection, the efficiency of VLPs was confirmed by qRT-PCR (**Figure 6B**). To assess local IgE-dependent mast cell degranulation by PCA reactions, the mice were intradermally injected with anti-DNP IgE into both ears and challenged intravenously with DNP-BSA and Evans blue 24 h later (**Figure 6A**). As presented in **Figures 6C, D**, Evans blue extravasation at PCA reaction sites was inhibited in mice injected with ORMDL3 overexpression VLPs, whereas, ORMDL3 knockdown VLPs and/or ATF6 knockdown VLPs injection enhanced Evans blue extravasation. As expected, the ORMDL3-OE + ATF6-KD group had higher Evans blue extravasation than the ORMDL3-OE group, which suggesting that ATF6 knockdown reversed the inhibited PCA reaction caused by ORMDL3 overexpression. Thus, we concluded that the ORMDL3-mediated ATF6 UPR pathway plays a crucial role in the degranulation of Ag-activated mast cells *in vivo*.

## Discussion

**Figure 7:**
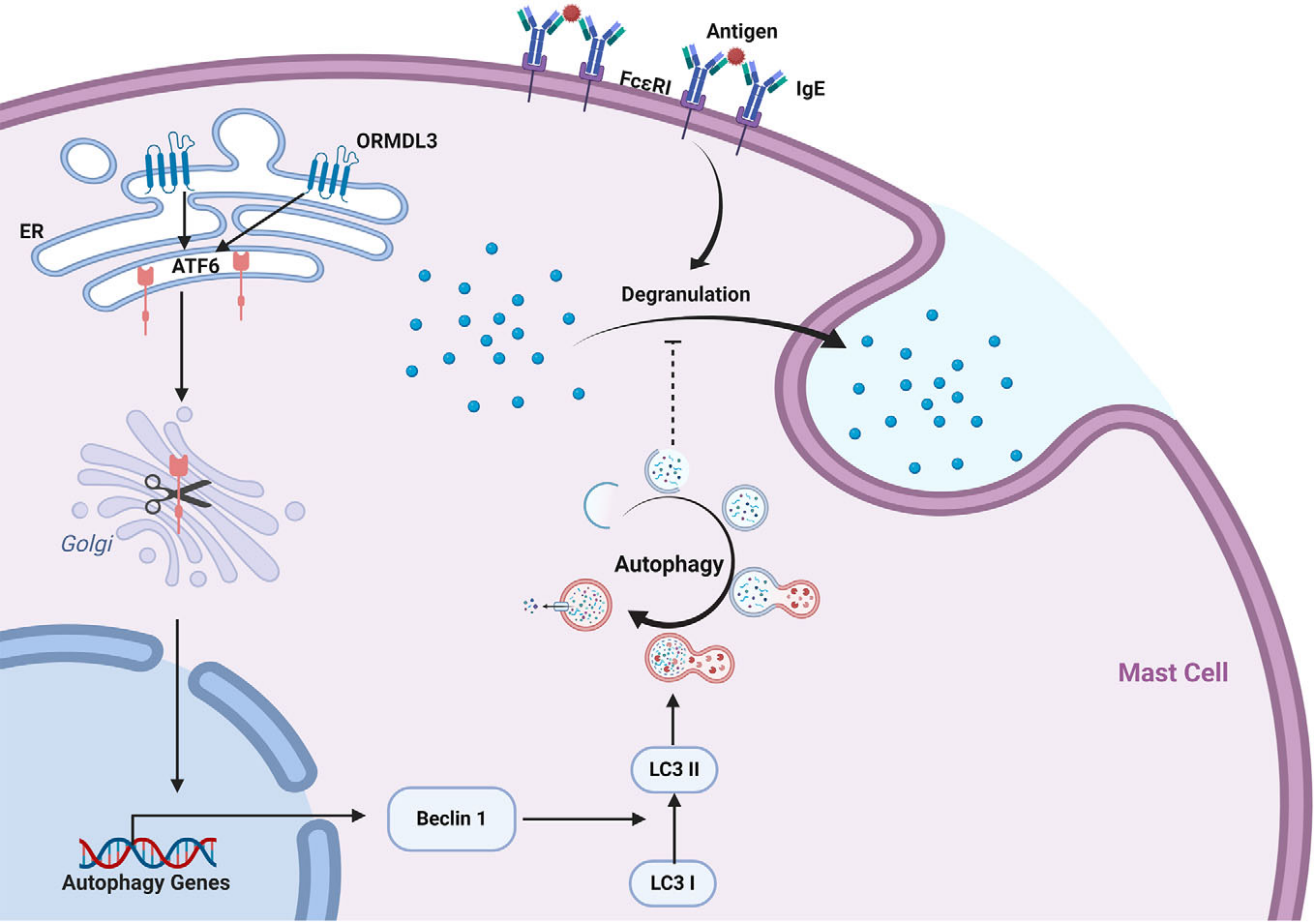
ORMDL3 negatively regulates mast cell activation *via* an ATF6-autophagy dependent pathway. ORMDL3 activates ATF6 pathway which upregulates the transcription of autophagy gene Beclin 1. This results in increased autophagic activity in mast cells. The FcϵRI-mediated mast cell activation can be inhibited by autophagy.

Our data revealed a novel mechanism of ORMDL3 mediated regulation of mast cell activation. We found that ORMDL3 expression was downregulated in Ag-activated MC/9 cells. Overexpression of ORMDL3 suppressed mast cell degranulation and attenuated the release of inflammatory cytokines and chemokines. The opposite responses were observed with the knockdown of ORMDL3 in mast cells. ORMDL3 overexpression also upregulated the ATF6-dependent UPR response as well as promoted autophagy in mast cells. Intriguingly, knockdown of ATF6 and/or autophagy inhibition repressed ORMDL3-mediated inhibition of mast cell activation. This was nicely corroborated with the findings that ORMDL3-ATF6-UPR suppressed PCA reaction. Taken together these data suggest that ORMDL3 negatively regulates mast cell activation and subsequent immune responses *via* ATF6-autophagy dependent pathway (**Figure 7**).

Mast cells are the crucial effector cells in IgE-dependent allergic diseases. Mast cells release various pro-inflammatory cytokines and chemokines upon antigen activation. We have shown that knockdown of ORMDL3 in Ag-activated mast cells increased mRNA expression of pro-inflammatory cytokines IL-6, IL-13 and TNF-α, and chemokines CCL3 and CCL4. This observation is consistent with a previous study using BMMCs in which the expression of cytokines and chemokines was found to be significantly enhanced in Ag-activated ORMDL3-KD BMMCs (23). Knockdown of ORMDL3 in Ag-activated mast cells causes enhanced phosphorylation of IκBα, a master regulator of transcription factor NF-κB (23, 30). ORMDL3 knockdown is also known to enhance nuclear localization of NF-κBp65 subunit which increases the transcription of NF-κB dependent genes of cytokines/chemokines including IL-6, IL-13, TNF-α, CCL3, and CCL4. However, Bugajev and colleagues reported that the mRNA expression of these cytokines/chemokines was higher in unstimulated ORMDL3-KD BMMCs whereas we did not find any difference in unstimulated cells (23). They also reported that ORMDL3 knockdown did not affect mast cell degranulation. This can be ascribed to the inherent differences in using cell lines versus primary cells and the different antigens being used in these experiments. It would be beneficial to perform a side-by-side comparison to investigate the underlying mechanisms of such differences.

ORMDL3 exhibits a negative regulatory role in Ag-activated mast cells, one that differs from activated eosinophils (9), airway smooth muscle (31), and bronchial airway epithelial cells (8). In fact, very few negative regulatory mediators have been reported in allergic diseases compared to positive mediators, therefore it is essential to explore the mechanisms involved. As an ER localized protein, the role of ORMDL3 in the regulation of ERS-induced UPR is an important mechanism to link ORMDL3 to asthma pathogenesis (5, 8, 32, 33). UPR has three major distinct arms: IRE1, PERK, and ATF6 (15, 16). Prior studies have demonstrated that the role of ORMDL3 in the regulation of UPR signaling is cell-specific. It is reported that the overexpression of ORMDL3 inhibited SERCA2b and activated the PERK/eIF2α arm of UPR in HEK 293 cells (human embryonic kidney cells) (22). In airway epithelium, ORMDL3 was found to activate the ATF6 pathway and subsequently regulate the expression of SERCA2b, which has been implicated in airway remodeling (8). In our study, we found that both SERCA2b and ATF6 expression was significantly increased in Ag-activated ORMDL3-OE cells, whereas it was decreased in Ag-activated ORMDL3-KD cells, which suggests that the ATF6 UPR pathway is regulated by ORMDL3 expression in Ag-activated mast cells. Meanwhile, our western blot showed that p-eIF2α (phosphorylated by PERK) was increased by overexpression of ORMDL3 and decreased by its knockdown. However, qRT-PCR analysis revealed that the mRNA levels of PERK were not significantly changed upon alteration of ORMDL3 expression, suggesting that ORMDL3 might regulate eIF2α expression independent of PERK, but this needs to be further investigated. To verify the contribution of the ATF6 UPR pathway to the regulatory role of ORMDL3 on mast cells, we constructed an ATF6-KD cell model in conjunction with ORMDL3 overexpression in which varied level of ATF6 knockdown was achieved using different concentrations of doxycycline. We observed that downregulation of ATF6 mRNA and protein level by 70% using 100 ng/ml Dox reversed the impairment of degranulation as well as cytokine and chemokine production by ORMDL3 overexpression. Although ATF6 mRNA and protein level were downregulated by 15% and 25% respectively using 10 ng/ml Dox, it did not reverse the inhibition effect of ORMDL3 on mast cell degranulation. However, it did partially restart cytokine and chemokine production, suggesting that cytokine and chemokine production is more responsive to the ORMDL3/ATF6 pathway regulation than degranulation. These results suggest a potential link between the ORMDL3 regulated ATF6 UPR pathway and Ag-mediated mast cell activation.

Following this we then aimed to investigate the vital molecular mechanisms by which the ORMDL3/ATF6 UPR pathway regulates mast cell activation. ORMDL3 is considered to be an autophagy-related gene, given its role in mediating inflammation, ERS and UPR, which in turn are stimulating factors that induce autophagy (19). It was suggested that high levels of ORMDL3 in B cells induce ERS through ATF6 and results in autophagy (34). In endothelial cells, oxidized low-density lipoprotein (ox-LDL) upregulates ORMDL3 expression and subsequently promotes autophagy (35). In macrophages, the anomalous expression of ORMDL3 affects autophagy and contributes to the risk of inflammatory diseases (21). Autophagy is a process that determines a cells fate in different ways (36); it can be regulated by any of the three branches of the UPR (ATF6, IRE1α, and PERK), although regulation is cell-specific and context-specific (12, 15–18). Interestingly, it is reported that autophagy plays a crucial role in mast cell degranulation. Conversion of LC3 I to LC3-II was found inherently induced in mast cells, and LC3-II localized in secretory granules of mast cells. Deletion of Atg7 has shown severe impairment of degranulation in BMMCs (29). Therefore, considering the following points that: there is a close relationship between ORMDL3 and autophagy; ORMDL3 regulates ERS-induced UPR; autophagy can be activated by the UPR pathway; autophagy is crucial to mast cell degranulation; we examined whether ORMDL3 may modulate autophagy through the ATF6 UPR pathway, thereby facilitating its regulatory role in mast cell activation. As expected, autophagy markers Beclin 1 and LC3B were shown to be increased in Ag-activated ORMDL3-OE cells, whereas they were decreased in ORMDL3-KD cells. Confocal microscopy was used to validate these results and demonstrated that ORMDL3-OE cells showed a strengthened formation of LC3B puncta, and ORMDL3-KD cells showed a decrease. ATF6 knockdown markedly reversed the upregulated autophagic activity by ORMDL3 overexpression, which confirmed that ORMDL3 regulates autophagy through ATF6. Furthermore, we demonstrated that inhibition of autophagy by 3-MA reversed the deficiency of degranulation and cytokine/chemokine production caused by ORMDL3 overexpression and an enhanced reversal was observed by ATF6 knockdown together with 3-MA treatment. Consequently, autophagy acts as a downstream effector of the ORMDL3/ATF6 UPR pathway.

The *in vivo* data corroborated the results obtained with MC/9 cells *in vitro*. PCA reaction is a commonly used measure of mast cell function *in vivo*. The reaction is induced by means of injecting antigen-specific IgE, which binds to FcϵRI on tissue mast cells and results in mast cells sensitization. Twenty-four hours later, antigen is co-administered intravenously with Evans blue dye, resulting in localized degranulation of mast cells, secretion of vasoactive mediators and extravasation of Evans blue (37, 38). The Evans blue extravasation was enhanced in mice injected with ORMDL3 knockdown VLPs and/or ATF6 knockdown VLPs, whereas it was inhibited in mice injected with ORMDL3 overexpression VLPs. Based on these data, we confirmed that the ORMDL3-mediated ATF6 UPR pathway negatively regulates the degranulation of Ag-activated mast cells *in vivo*. One limitation of our study is that we did not use *Ormdl3* ^−/−^ mice. However, it is important to note that these mice have elevated serum levels of total sphingolipids, including ceramides (13), which binds to leukocyte mono-immunoglobulin-like receptor 3 (LMIR3) and inhibits mast cell activation (39, 40). Another limitation of the study is the use of single cell line (MC/9) for all the experiments. MC/9 cell line is derived from the fetal liver of a F1 mice. The size and morphology of MC/9 are similar to those of BMMCs. MC/9 expresses functional FcϵRI and CD117. Its binding ability to mouse IgE is similar to that of mouse peritoneal mast cells. In our experiment, we successfully transduced MC/9 with ORMDL3 and ATF6 virus like particles (VLPs). We have tested mouse BMMCs for this investigation. It was practically difficult to obtain large number of cells required for this study. Moreover, we could not achieve satisfactory transduction efficiency of BMMCs which was critical for the investigation of the role of ORMDL3/ATF6 in mast cell activation.

In conclusion, our work supports the current hypothesis that ORMDL3 acts as a negative modulator of mast cell activation, further to this we have identified that the ATF6 UPR-autophagy pathway is critical in this response. A limitation of our study is that we did not identify the mechanism by which autophagy affects Ag-mediated mast cell activation. Although a previous study found that cellular ATP levels, which may be crucial for activation of kinases involved in mast cell function, were increased in Atg7^−/−^ BMMCs (29), whether autophagy participates in mast cell function by alteration of cellular ATP levels is currently unknown and needs to be further investigated. Nevertheless, our findings provide evidence of the negative regulatory role of ORMDL3 on mast cell degranulation as well as cytokine and chemokine production. Additionally, we demonstrated that ORMDL3 selectively regulates the ATF6 UPR-autophagy signaling pathway, which is an important mechanism to link ORMDL3 to mast cell physiology providing a cellular and molecular explanation for the association between ORMDL3 and asthma pathogenesis.

## Data Availability Statement

The original contributions presented in the study are included in the article/**Supplementary Material**. Further inquiries can be directed to the corresponding author.

## Ethics Statement

The animal study was reviewed and approved by the animal ethics committee of Shanghai General Hospital.

## Author Contributions

JL designed and performed experiments, analyzed and interpreted the data, and wrote the manuscript. MU and HJ designed experiments, analyzed and interpreted the data, and edited the manuscript. YTL, LLin, JW, XP, HL, YNL, and YG performed experiments. LLi designed experiments, supervised the work, contributed to study design and data interpretation, and edited the paper. All authors contributed to the article and approved the submitted version.

## Funding

This work was supported by the National Natural Science Foundation of China (grant no. 81871267, 81971509, 81302551) and Shanghai Shen Kang Hospital Development Center (16CR3098B). JL was supported by a joint PhD scholarship, China Scholarship Council (CSC).

## Conflict of Interest

The authors declare that the research was conducted in the absence of any commercial or financial relationships that could be construed as a potential conflict of interest.
