# Supplementary material for: ORMDL3 Functions as a Negative Regulator of Antigen-Mediated Mast Cell Activation via an ATF6-UPR-Autophagy–Dependent Pathway
Source: Front Immunol. 2021 Feb 19;12:604974. doi: 10.3389/fimmu.2021.604974 (PMC7933793; doi:10.3389/fimmu.2021.604974)
Supplement: Supplementary file 1 [file DataSheet_1.pdf]

Supplementary Material

Supplementary Figures

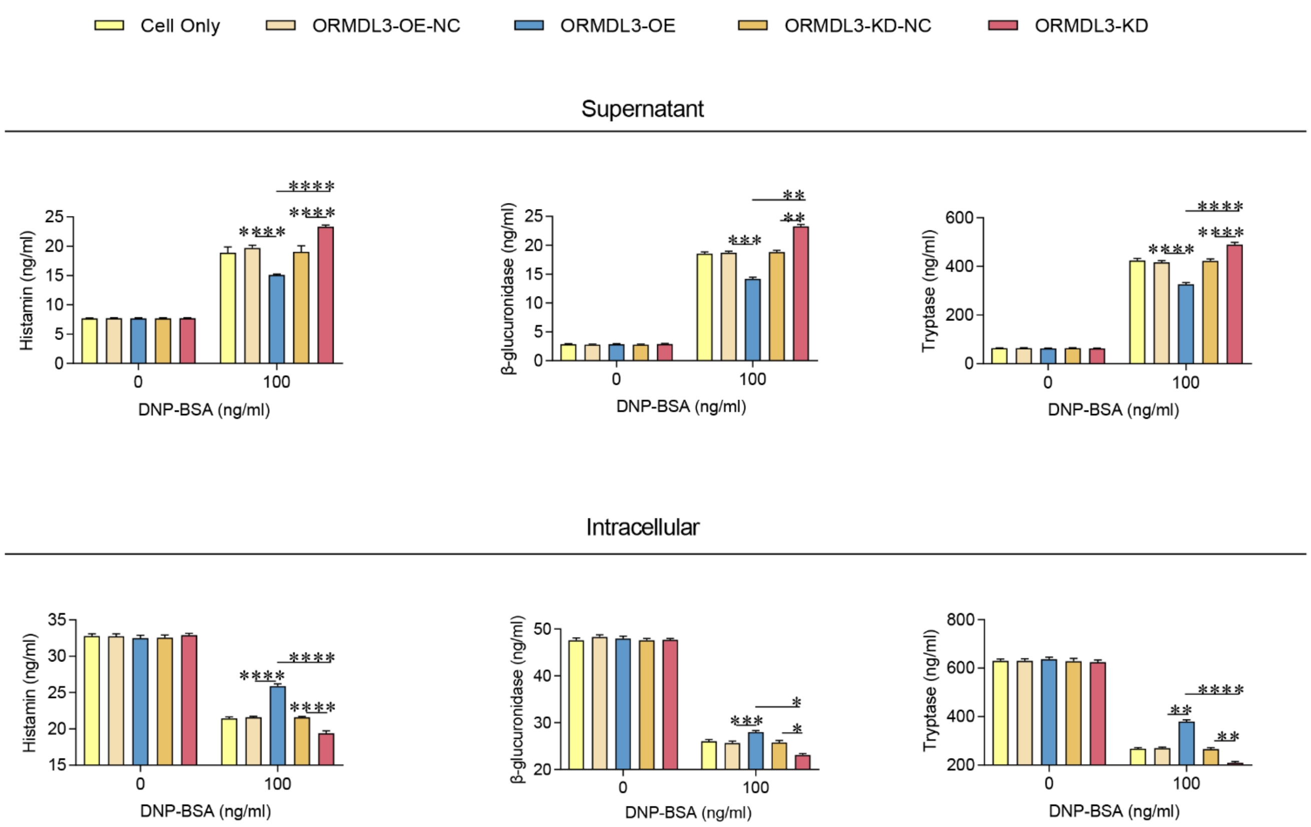

Supplementary Figure 1. ORMDL3 negatively regulates degranulation in antigen-activated mast cells. MC/9 cells were transduced with VLPs conveying ORMDL3-shRNA and ORMDL3 and referred to as ORMDL3-KD and ORMDL3-OE respectively. Controls transduced with empty VLPs are referred to as ORMDL3-KD-NC and ORMDL3-OE-NC. Cells were sensitized overnight with 1 µg/ml of anti-DNP mouse IgE followed by stimulation with PBS (0 ng/ml DNP-BSA) or 100 ng/ml of DNP-BSA for 30 min. The concentrations of histamine, β-glucuronidase and tryptase in both supernatant and cell lysates were measured by ELISA. Results are shown as mean ± SDs of 3 independent experiments. *$P < 0.05$; **$P < 0.01$; ***$P < 0.001$; ****$P < 0.0001$. OE, overexpression; KD, knockdown; NC, negative control.

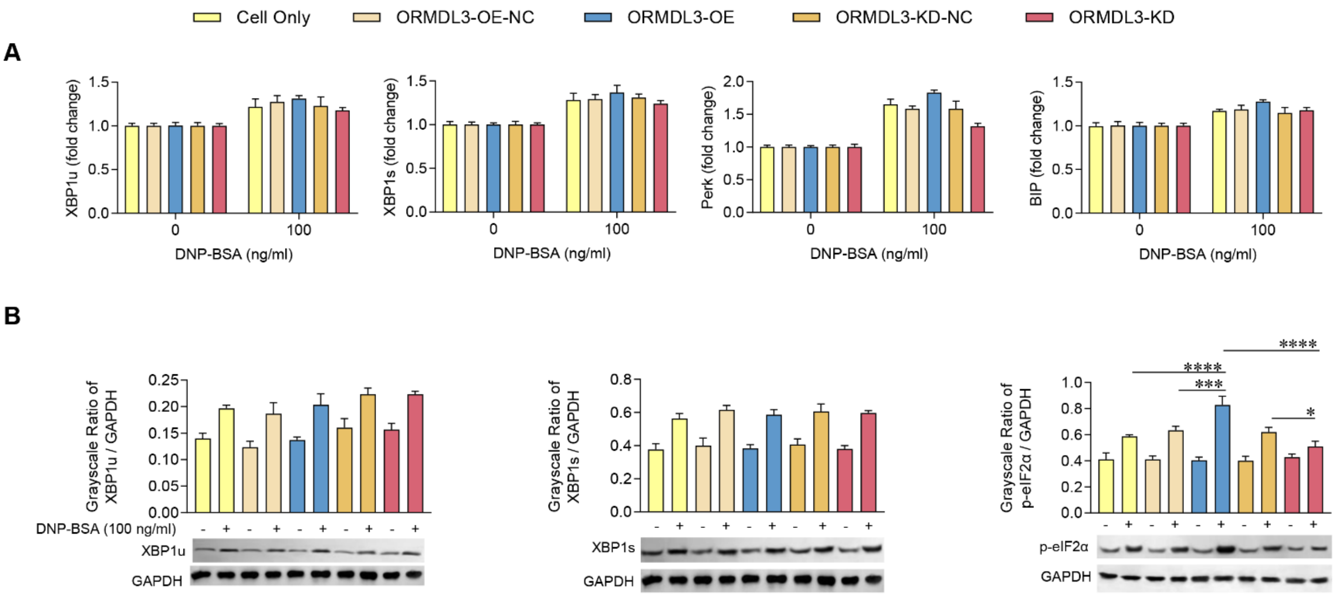

Supplementary Figure 2. Effects of enhanced or reduced expression of ORMDL3 on IRE1 and PERK UPR pathways in mast cells. MC/9 cells were treated as described in Supplementary Figure 1. **(A)** qRT-PCR quantification of mRNAs encoding XBP1u, XBP1s, Perk and BIP in non-activated and Ag-activated ORMDL3-KD, ORMDL3-OE cells and their corresponding controls. **(B)** Western blot analysis of protein levels of XBP1u, XBP1s and p-eIF2α in non-activated and Ag-activated ORMDL3-KD, ORMDL3-OE cells and their corresponding controls. Results are shown as mean ± SDs of 3 independent experiments. **P* < 0.05; ****P* < 0.001; *****P* < 0.0001. OE, overexpression; KD, knockdown; NC, negative control.

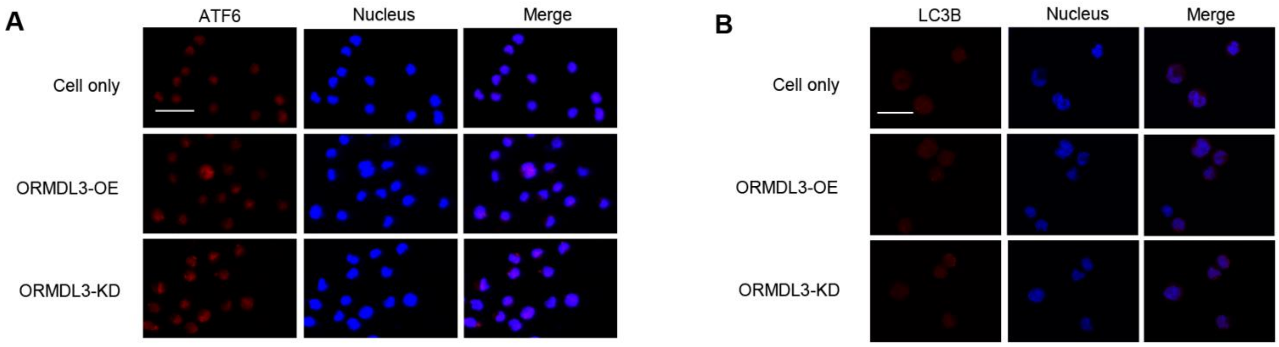

Supplementary Figure 3. Enhanced or reduced expression of ORMDL3 does not affect ATF6-UPR and autophagy in non-activated mast cells. MC/9 cells were treated as described in Supplementary Figure 1. Confocal images depicting the expression of ATF6 **(A**, red**)** and LC3B **(B**, red**)** in non-activated mast cells. Nuclei (blue) were stained with DAPI. Scale bar 25 μm. Results are shown as mean ± SDs of 3 independent experiments. OE, overexpression; KD, knockdown.

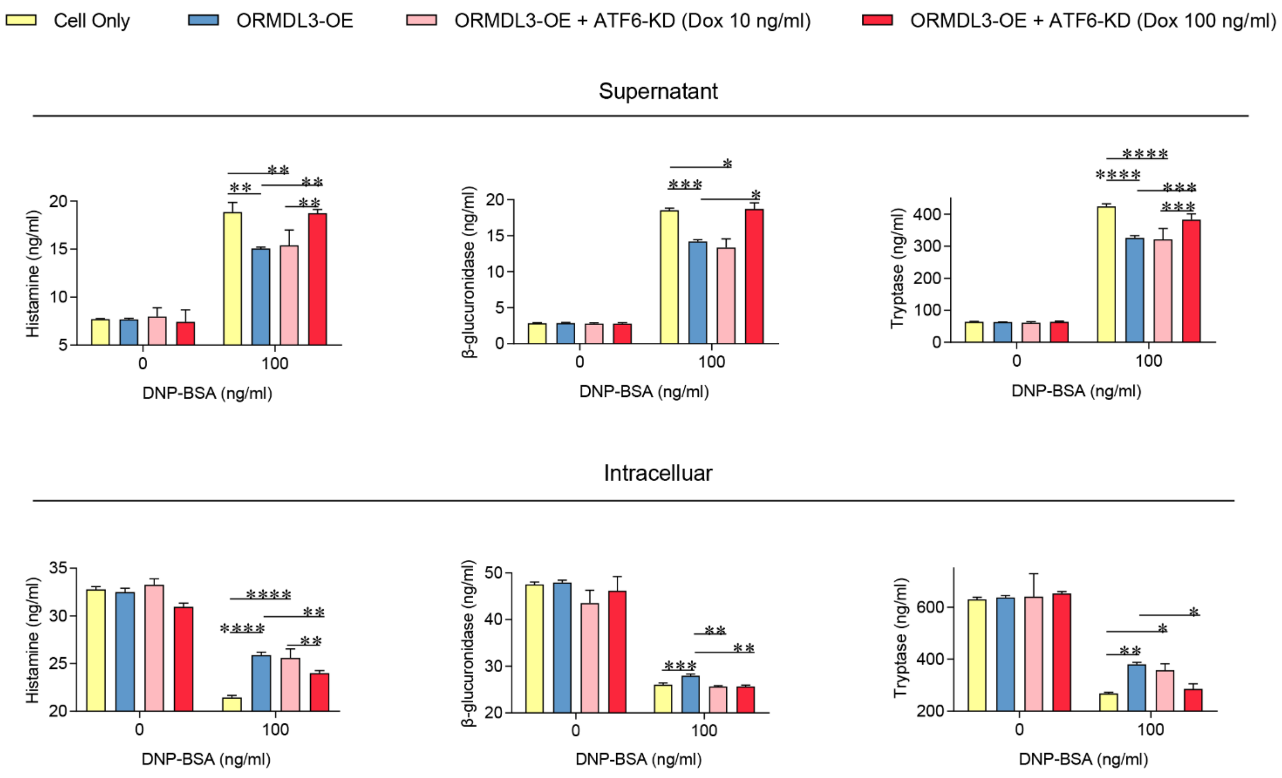

Supplementary Figure 4. Inhibition of ATF6 UPR reverses ORMDL3 overexpression-mediated suppression of mast cell degranulation. Knockdown of ATF6 was conducted by transducing ORMDL3-OE cells with VLPs conveying ATF6-shRNA and the addition of doxycycline (Dox, 10 ng/ml or 100 ng/ml). Cells were sensitized overnight with 1 μg/ml of anti-DNP mouse IgE followed by stimulation with PBS (0 ng/ml DNP-BSA) or 100 ng/ml of DNP-BSA for 30 min. The concentrations of histamine, β-glucuronidase and tryptase in both supernatant and cell lysates were measured by ELISA. Results are shown as mean ± SDs of 3 independent experiments. *P < 0.05; **P < 0.01; ***P < 0.001; ****P < 0.0001. OE, overexpression; KD, knockdown.

Supplementary Table 1. Primer sequences used in qRT-PCR

Gene (mouse)	Forward primer (5'→3')	Reverse primer (5'→3')	Amplicon size (bp)
ORMDL3	GTGAGGTGAACCCCAACACA	GTACATGCCCAGGTTGTGGA	161
IL-6	GCCTTCTTGGGACTGATG	AGGTCTGTTGGGAGTGGTA	96
IL-13	TGGTTTGCTGCCTATGCCCT	GCTACAGTGAGGTAGCAGAGTTA	101
TNF-α	GAGAGTGGTCAGGTTGCCTC	GCACCTCAGGGAAGAATCTGG	94
CCL3	CAGCCAGGTGTCATTTTCCTGA	ATGCAGGTGGCAGGAATGTT	196
CCL4	AAACCTAACCCCGAGCAACA	CTTTTGGTCAGGAATACCACAGC	228
ATF6	GAACTTCGAGGCTGGGTTCA	CTTGCAGCTCACTCCCAGAA	184
XBP1u	TCAGACTATGTGCACCTCTGC	TTGTCCAGAATGCCCAAAAGG	129
XBP1s	AGAGCTGGGCATCTCAAACC	GGAGAAGGGGAGCCCTCATA	105
BiP	TCGACTTGGGGACCACCTAT	AGTGAAGGCCACATACGACG	110
PERK	GCCGACGATCAAATGGAAGC	ACCTGACTGTGATCTGCGTG	221
Actin	AGAGGGAAATCGTGCGTGAC	CCATACCCAAGAAGGAAGGCT	112
Beclin 1	AGCTGGAGTTGGATGACGAAC	GATTGTGCCAAACTGTCCGC	137
SERCA2b	CCTTTGCCGCTCATTTTCC	TGTGCTGTAGACCCAGACCA	252
LC3B	TCTGAGCAGAGGGAAAAGGG	CTCGCTCATGTTCACGTGGT	118
